# Monitoring county-level chlamydia incidence in Texas, 2004 – 2005: application of empirical Bayesian smoothing and Exploratory Spatial Data Analysis (ESDA) methods

**DOI:** 10.1186/1476-072X-8-12

**Published:** 2009-02-26

**Authors:** Kwame Owusu-Edusei, Chantelle J Owens

**Affiliations:** 1 Division of STD Prevention, Centers for Disease Control and Prevention, 1600 Clifton Road MS E-80, Atlanta, GA 30333, USA

## Abstract

**Background:**

Chlamydia continues to be the most prevalent disease in the United States. Effective spatial monitoring of chlamydia incidence is important for successful implementation of control and prevention programs. The objective of this study is to apply Bayesian smoothing and exploratory spatial data analysis (ESDA) methods to monitor Texas county-level chlamydia incidence rates by examining spatiotemporal patterns. We used county-level data on chlamydia incidence (for all ages, gender and races) from the National Electronic Telecommunications System for Surveillance (NETSS) for 2004 and 2005.

**Results:**

Bayesian-smoothed chlamydia incidence rates were spatially dependent both in levels and in relative changes. Erath county had significantly (p < 0.05) higher smoothed rates (> 300 cases per 100,000 residents) than its contiguous neighbors (195 or less) in both years. Gaines county experienced the highest relative increase in smoothed rates (173% – 139 to 379). The relative change in smoothed chlamydia rates in Newton county was significantly (p < 0.05) higher than its contiguous neighbors.

**Conclusion:**

Bayesian smoothing and ESDA methods can assist programs in using chlamydia surveillance data to identify outliers, as well as relevant changes in chlamydia incidence in specific geographic units. Secondly, it may also indirectly help in assessing existing differences and changes in chlamydia surveillance systems over time.

## Introduction

Chlamydia is the most prevalent reportable disease in the United States with an estimated 2.8 million cases each year [1,2]. Untreated chlamydial infections in women have been associated with more serious reproductive complications such as pelvic inflammatory disease (PID), ectopic pregnancy, tubal infertility, and chronic pelvic pain [3-6]. In men, chlamydia has been associated with urethritis and other complications such as epididymitis and acute proctitis [7-9]. Thus, it is a public health problem that has attracted public attention, albeit not as much as would be desired.

Several previous studies have recommended that the design and implementation of effective interventions to control or prevent sexually transmitted diseases (STDs) should be grounded on a good understanding of the existing and emerging spatiotemporal patterns because STDs are characterized by geographic patterns [10-16]. An emerging approach to achieving this end is the application of Exploratory Spatial Data Analysis (ESDA) methods which draws from the field of spatial statistics [17]. At the state-level, ESDA methods can be used by state health officials to monitor spatial and temporal variations in rates using counties as spatial units. ESDA can also assist in identifying and monitoring hot spots ("problem counties") that may not be obvious otherwise. These methods can aid health officials to design more location-specific prevention programs that take into account global and local spatial influences. It is also valuable to be able to assess and develop surveillance systems that can immediately and effectively pick up warning signs of increases in any particular STD. The ideas and motivation for the application of these methods to STD were drawn from pioneering works in the area of ESDA by Luc Anselin and others on juvenile crime and cancer rates, among others [18-21].

The primary objective of this study was to use ESDA methods to identify and monitor Bayesian-smoothed chlamydia incidence rates using county-level data from the state of Texas. Our choice of counties as the unit of analysis was based on availability of data. Finer spatial units (cities or census tracts) may provide more location-specific information that can inform the design and implementation stages of existing or future interventions. Majority of chlamydia cases are asymptomatic prompting recommendations for routine screening for young women by individuals and organizations [22-30]. In view of this, differences in the incidence rates may be the result of differences in existing surveillance systems. Thus, indirectly, ESDA may help to identify disparities in chlamydia surveillance systems.

## Methods

### Data

Data used in this study was obtained from the National Electronic Telecommunications System for Surveillance (NETSS) which is maintained by the Centers for Disease Control and Prevention (CDC). We used the overall incidence rates (per 100,000 residents, for all race, sex and age groups) for each county provided by the surveillance system.

### Spatial relationship concept

We used the standardized 1^st^- order Queen Neighbors (all counties that share a border with the referent county) as the criteria for identifying neighbors. Spatial relationship through out this study was executed by the use of a spatial weight matrix.

### Empirical Bayesian smoothing

Raw rates derived from different counties across a region may result in unstable rates because of the small number of cases from small population base counties. The corollary to this is that the rates may not fully represent the relative magnitude of the underlying risks if compared with other counties with high population base. To reduce this, empirical Bayesian smoothing, which was proposed by Clayton and Kaldor [31] was applied to the computed raw rates. The formular for the empirical Bayesian smoothing is Ŕ = μ + ś(r - μ), where Ŕ is the new smoothed rate estimate, μ is the global population-weighted mean, ś is the shrinkage factor, and r is the level incidence rate (see Waller and Gotway [32] for more details). We used the global smoothing method which computes the rates using the global mean (as against the local mean) of the rates because it was a better smoother. It also reduced the likelihood of concluding that there was clustering. Thirdly, we used the state global mean for smoothing because this was conducted from the state's perspective. Thus, the frame of reference was the average rate across all the counties in the state of Texas. However, for any particular region within the state, one may use a regional global mean for smoothing and then use the local Moran's I to identify local outliers. Empirical Bayesian smoothing forced the rates towards the center (average) and increased the likelihood of clustering. However, it served as an additional confirmatory indicator for identifying outliers. This was because if after forcing the rates to be more alike, some were still outliers, then those smoothed county rates were true outliers. Table 1 shows summary statistics of the smoothed rates for all 254 counties. All the analyses were done using the Bayesian-smoothed rates (including those reported). The statistic for outliers was the computed z-values, which was the difference between the observed and expected mean of the smoothed rates standardized by the standard deviation. Thus it had a mean of zero and a variance of 1.

**Table 1:** Summary statistics of Bayesian-smoothed chlamydia incidence rates (n = 254)

**Year**	**Mean**	**Standard Deviation**	**Minimum**	**Maximum**
2004	251	117	84	778
2005	252	130	60	1126

### Measuring spatial dependence

We used Moran's I [33], a statistical test for global spatial autocorrelation (dependence) in group-level data to identify departures from spatial randomness revealing the existing spatial patterns, such as clusters. The hypotheses for this test are:

Null: Smoothed rates in different regions are spatially independent,

Alternative: Smoothed rates are not spatially independent.

If the resulting value was positive, then there was spatial autocorrelation – nearby areas had similar rates, indicating global spatial clustering. Conversely, if the value was negative, then nearby areas were dissimilar. A value close to zero indicates random spatial units. We used Anselin's Local Moran test which was an extension of Moran's test to identify local spatial autocorrelation [18]. This test was used to identify local outliers by comparing counties to their contiguous counties – how different the rates were for any spatial unit (county in this case) from its immediate neighbors.

After computing the appropriate statistic from the smoothed rates, a Monte Carlo Randomization (MCR) procedure was used to recalculate the statistic from the randomized data observations to generate a reference distribution using 999 permutations. The p-values were computed by comparing the observed statistic to the distribution generated by the MCR process. We used Simes Correction [34] to adjust the p-values to account for the lack of independence in the statistics computed. We used GeoDa (version 0.9.5-i) software application by Luc Anselin, 2004.

The basic steps in ESDA used in this study were:

1. Mapped the Bayesian-smoothed rates in classes for preliminary visual analysis – identified outliers and spatial association.

2. Conducted a statistical test to confirm or reject spatial dependence, and

3. Computed local Moran statistics to map local outliers.

We also analyzed trends by computing relative changes (from 2004 to 2005) for each county to identify global and local outliers using the same steps outlined above.

## Results

**Figure 1:**
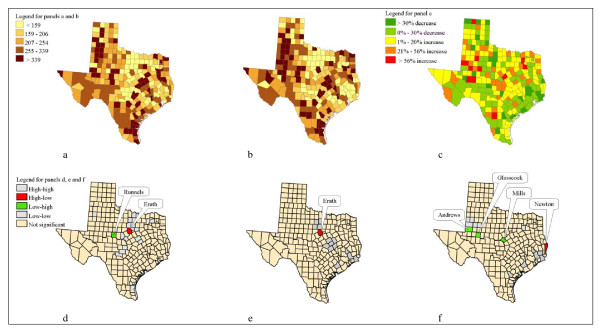
**Bayesian-smoothed chlamydia rates in Texas by county, 2004–2005 showing local Moran tests**. a. Graduated color scheme map showing Bayesian-smoothed chlamydia rates for 2004; b. Graduated color scheme map showing Bayesian-smoothed chlamydia rates for 2005; c. Graduated color scheme map showing percent change (2004 to 2005) in Bayesian-smoothed chlamydia rates; d. Local Moran significance map of chlamydia rates for 2004; e. Local Moran significance map of chlamydia rates for 2005; f. Local Moran significance map for percent change in chlamydia rates from 2004 to 2005.

Categorical maps for the 2004 and 2005 global empirical Bayesian-smoothed rates are presented in Figure 1 (panels a and b, respectively) using the same ranges. Outliers (ten counties with the highest rates) of the smoothed rates for the two years are presented in Table 2, together with associated z-value. Bell county had rates that were 4.5 and 4.7 standard deviations from the mean for 2004 and 2005, respectively. All the ten counties with the highest rates are at least 1.81 standard deviations from the mean for 2004 and 2005. Six counties were consistently among the highest ten of the smoothed rates for both years: Bell, Falls, Potter, Taylor, Kleberg, and Lubbock counties (Table 2). This indicated that they were true outliers for chlamydia incidence. Rains, Rockwall and Chambers counties also were found in the lowest ten for the two years (not shown in table).

**Table 2:** Bayesian-smoothed chlamydia incidence rates among the ten counties with the highest incidence in Texas, 2004 and 2005

**2004**			**2005**			**Relative change 2004–2005**		
**County**	**Rate**	**Z-value**	**County**	**Rate**	**Z-value**	**County**	**% change**	**Z-value**

Bell	778	4.50	Falls	1126	6.72	Gaines	173	3.71
Falls	740	4.18	Bell	866	4.72	Hunt	99	2.56
Hale	679	3.65	Potter	668	3.20	La Salle	97	2.52
Potter	652	3.42	Kleberg	614	2.78	Hamilton	79	2.17
Nolan	568	2.70	Hays	539	2.20	Sherman	72	2.03
Taylor	563	2.66	La Salle	524	2.09	Martin	71	2.01
Kleberg	561	2.65	Gregg	505	1.94	Garza	70	1.99
Lubbock	519	2.29	Lubbock	503	1.93	Van Zandt	69	1.95
McLennan	517	2.27	Taylor	501	1.92	Mitchell	66	1.90
Coke	513	2.16	Bexar	487	1.81	Upton	64	1.85

A visual inspection of Figure 1 (panels a and b) gives an indication of spatial association – counties with similar color shades had the tendency to be near each other. We carried out the formal statistical test using Moran's I. The test statistics were 0.19 (p < 0.01) and 0.21 (p < 0.01) for 2004 and 2005, respectively. Thus we rejected the null of spatial independence and concluded that there was sufficient evidence of spatial dependence.

The local Moran significance maps (Figure 1, panels d and e) indicated that in both years, smoothed chlamydia incidence rates for one county (Erath county) was significantly (p < 0.05) higher than its contiguous neighbors. Erath county had smoothed rates of more than 300, while its neighbors had 195 or less in the two years examined. For 2004, the smoothed rates for Runnels county was significantly (p < 0.05) lower than its immediate neighbors (see Figure 1, panel d). The rest of the rates for the other counties were either similar (high-high or low-low) to its neighbors or not statistically different from them.

### Temporal patterns

To examine temporal patterns, we computed the relative changes (percent changes) in the smoothed rates from 2004 to 2005. Figure 1, panel c shows a graduated scheme map of the relative change in chlamydia rates. Our formal test also indicated evidence of spatial dependence in the relative changes that occurred from 2004 to 2005 (0.14, p < 0.01).

The highest ten values of relative change in the smoothed rates are also presented in Table 2. Gaines county had the highest relative change. The chlamydia rates for Gaines county increased from 139 to 379 (cases per 100, 000 residents); a 173 percent increase. Except for Gaines county all the other nine counties had z-values within 3 standard deviations of the mean.

### Local Moran significance maps

Figure 1, panel f shows local Moran significance maps used to identify changes in smoothed rates for counties that were significantly higher or lower than their neighbors. Percent change in Newton county (15% increase) was significantly (p < 0.05) higher than its contiguous counties (Orange, 54% decrease; Jasper, 6% increase; Sabine, 37% decrease). The relative changes in smoothed rates for Andrews, Glasscock and Mills counties were significantly (p < 0.05) lower than their contiguous counties. The rest were either similar to their immediate neighbors, or not significantly different.

## Discussion

We applied empirical Bayesian smoothing and ESDA methods in GIS to study the most commonly reported STD in the United States – chlamydia. County-level chlamydia incidence rates for 2004 and 2005 in the state of Texas were used to characterize spatiotemporal patterns. Based on data from the National Electronic Telecommunications System for Surveillance (NETSS), our results indicated that empirical Bayesian-smoothed chlamydia rates were spatially dependent for the two years examined. Furthermore, the relative changes that occurred between 2004 and 2005 were also spatially dependent. Six counties (Bell, Falls, Potter, Taylor, Kleberg, and Lubbock) were among the highest ten counties for the two years examined. Also, Erath county had significantly (p < 0.05) higher smoothed rates than its immediate neighbors for the two years examined. The highest relative increase in chlamydia rate was in Gaines county, which experienced over 170% increase in smoothed rates. However, the increase in chlamydia rate in Gaines county was not significantly different from its contiguous neighbors. Relative change in chlamydia rates in Newton county was significantly (p < 0.05) higher than its contiguous counties. The counties identified suggest that they should be considered as the targets for further appraisal. Thus, more detailed examination of the data is required for these counties.

### Limitations

Surveillance data are not perfect. However, for chlamydia, the problem may be more pronounced. Most of chlamydia cases are asymptomatic so the data on incidence may largely be based on adherence to screening recommendations by individuals and organizations [22-30] that vary from county to county and from group to group. Nonetheless, the strength in this type of analyses is that it has the potential to prompt health officials to investigate the data further and subsequently help identify the disparities in the existing screening patterns. For instance, it is possible that Erath county has a relatively better chlamydia screening program compared to its neighbors, or that Gaines county substantially increased screening from 2004 to 2005.

One of the limitations of the Bayesian empirical smoothing method used in this study is that it can potentially overestimate the test statistic for spatial dependence. However, the conclusion reached (i.e., existence of spatial dependence) in this study was the same as in previous studies in which different smoothing methods and tests were used. Previous studies found spatial dependence in chlamydia rates using formal statistical tests on county-level data for 2000 from Texas [35] and 2000–2002 data on census blocks from Richmond, Virginia [36]. Our review of the literature did not provide any information on the application of a formal test for spatial dependence on the relative changes of chlamydia rates. Thus more studies should be conducted to study the existence of spatial dependence of the relative changes in chlamydia rates.

By default, focusing on any particular spatial jurisdiction, such as a state, precludes one from studying the effects of spatial association with contiguous counties in neighboring states. One limitation of this study was that analyses of border counties did not include spatial effects from the counties in the bordering state. As an example, Newton County which was on the border with Louisiana would have to be examined closely to understand the pattern discovered in this study. Therefore, there was the need to further investigate the spatial relationships that existed for outliers that were situated on the border of the state, as their rates may be the result of interaction with counties in the states bordering them that may have been ignored.

## Conclusion

The methods used in this study can be applied to any state, county or city, and for any age group within the chosen spatial/geographic unit. However, where data is available, smaller geographic units are preferable in such analyses. ESDA is one of the methods available for monitoring diseases. There are also other smoothing methods available in the literature, but there are no reports on a comprehensive objective assessment of all the available methods. Therefore, further research is needed in this area. The use of two or more methods on the same dataset may enhance validity if the final results are robust. Additionally, as shown in this study, simple mapping for any geographic units of interest by classes and by associated changes overtime, followed by critical inspection may help to correctly describe the existing spatiotemporal patterns. Identifying and describing the patterns can guide the design and implementation stages of interventions/programs, as well as indirectly help to evaluate existing chlamydia surveillance systems.

## Competing interests

The authors declare that they have no competing interests.

## Authors' contributions

KOE conceptualized the ideas of the study and drafted the manuscript. CJO assisted with data organization and analyses. All authors helped to interpret results, reviewed and approved the final manuscript.
